# 

**Published:** 1915-09-18

**Authors:** 


### A RED CROSS ANNIVERSARY.

Saturday, October 21, is the anniversary of the amalgamation for war purposes of the British Red Cross Society and the Order of St. John. The Joint Committee has decided to utilise that day for a special appeal throughout the British Isles and the Oversea Dominions on behalf of sick and wounded sailors and soldiers. A great effort will be made to raise funds which will be specially devoted to the needs of the sick and wounded. As the day approaches we hope to say more about the matter.

### Buildings Contemplated and in Progress.

ANNAN.—It is understood that a new hospital, with accommodation for at least 500 beds, is about to be built at Annan. The site selected is near to the Annan Combination Hospital, Bellsprings Road.

BATH.—The site for the proposed new military hospital to be erected at Coombe Park, Bath, has been pegged out preparatory to making a plan, which will probably comprise a range of huts.

CYMLA (NEATH).—The Joint Hospital Committee is inviting tenders for the erection of a new joint isolation hospital at Cymla, Neath. The plans comprise administrative block, porter's lodge, diphtheria block, scarlet-fever block, enteric block, discharging block, and laundry block. These plans, together with specifications and bills of quantities, may be seen at the offices of the architect, Mr. J. C. Rees, M.S.A., Parade Chambers, Neath. Tenders are to be delivered to Mr. E. Powell, solicitor, Walter Street, Neath, by October 1.

EDMONTON.—The Guardians have approved a scheme for the extension of the Edmonton Military Hospital by 300 beds accommodated in huts. The cost of the scheme is estimated at £13,000, including £4,000 for furniture, etc.

GALEMIRE.—It has been decided by the Galemire Joint Hospital Committee, Galemire, Whitehaven, to obtain competitive designs for a small hospital. Particulars may be obtained from the Clerk, Mr. E. B. Croasdale, Union Hall, Whitehaven.

GOSPORT.—The building of the new Haslar Military Hospital at Gosport, Hants, has now been commenced. The contractor is Mr. J. Hart, South Wharf, Cleveland Road, Gosport.

TYRONE.—The Co. Tyrone Tuberculosis Committee has decided to accept tenders for converting Dungannon House for the purpose of a county sanatorium. The cost, including new pavilions to be erected in the grounds, is estimated at £4,765.

### The New Zealand Hospital.

ACCORDING to the *British Australasian*, plans have been made for the new buildings for the New Zealand Hospital, and it has been decided that these shall not be exactly on the plan of the open-air base hospital at Cambridge, though all the best features of the Cambridge Hospital will be copied. Two blocks are to be erected of asbestos sheets. They will be capable of housing a hundred beds each, and giving ample accommodation for kitchen departments, etc. The new wing of the New Zealand Hospital will have windows all along the front, which may be taken out altogether or kept very wide open.

### Medical Appointments.

Colonel D. J. Mackintosh, M.V.O., Medical Superintendent of the Western Infirmary, Glasgow, and Assistant Director of Medical Services, 2/1st Lowland Division, in addition to his other duties, was some months ago appointed to supervise the administration and organisation of all military, war, and Territorial general hospitals in the Glasgow area.

Mr. J. Stanislaus Lafferty, L.D.S., R.C.S., has been appointed hon. dental surgeon, and R. A. Parsons, M.D., has been appointed anæsthetist to the Male and Female Lock Hospitals.

Dr. H. D. A. Spence has been appointed house surgeon to the London Lock Hospital (male department), Soho, and Miss Courtauld, M.D. Brux., M.R.C.S., has been appointed house surgeon to the Female Lock Hospital, Harrow Road.

The following appointments to the resident staff of the Bradford Infirmary have been made by the Board of Management : Mr. H. A. Rippiner, M.B., Ch.B. (Edin.), at present house surgeon, to be resident surgical officer; Mr. G. Paterson Burr, M.B., Ch.B. (Aber.), at present house physician, to be house surgeon; Mr. Irvine McDowall, M.B., Ch.B. (Edin.), to be house physician.

### Personalia.

Mr. A. S. J. M. Huggett has won the first scholarship, value £150, and Mr. H. S. Le Marquand the second, value £60, of the Entrance Science Scholarships, 1915-16, to St. Thomas's Hospital Medical and Surgical College.

Mr. G. B. Stephenson, of Easby Road, Bradford, has given a donation of fifty guineas as a special birthday gift to the Bradford Royal Infirmary. The Board of Management has appointed Mr. Stephenson a vice-president of the institution.

### Norfolk War Hospital.

As an annexe to the Norfolk War Hospital a spacious building has been acquired in the vicinity and has already received a hundred and seventy wounded men. It is interesting to note that the Norfolk War Hospital has just received an added dignity and at the same time a considerable addition to its responsibilities by being made the Central Hospital for the county. All the V.A.D. hospitals in the county come into official relations to it and have to notify to it the number of their vacancies, and not to Colchester, as was formerly the case. Medical Boards for invaliding men out of the Service now sit within its walls, and not at Colchester or at Norwich Barracks. A great deal of other administrative work relating to the V.A.D. hospitals of the county now goes to Thorpe, and they are each subject to a monthly inspection by an officer who has been newly appointed to the staff for that purpose.

### A NEW RED CROSS HOSPITAL.

The City branch of the British Red Cross Society has recently acquired Buecker's Hotel, Finsbury Square, as a hospital for wounded soldiers. The arrangements for opening the building are nearly complete, and it is expected that the first patients will be received within the next few days. The hospital will accommodate 100 men.

### Gifts and Bequests.

The Hon. Maude Alethea Stanley, of Westminster, S.W., daughter of the second Lord Stanley of Alderley, who started the first club for working girls in Greek Street, Soho, in 1880, has left £450 to the New Hospital for Women, Euston Road, and £500 to the Girls' Club and Home, Soho.

The London Lock Hospital has received £2,000 from the executors and trustees of the late Sir William Dunn. It has also received the following gifts to the emergency appeal now being issued: "E. F.," £100; Mrs. Mappin, £50; Mrs. Mackinnon, £20; Mrs. Middleton, £20; and to the male hospital building fund deficit, £50 from Francis J. Meynell, Esq.

Mrs. Betsy Ridge, of Ealing Common, bequeathed £500 to the Ealing Cottage Hospital; £200 each to the British Home for Incurables, Streatham, and the Royal Hospital for Incurables, Putney; £100 each to the North London Hospital for Consumption, Hampstead, the Metropolitan Hospital, London Fever Hospital, Islington, St. Mary's Hospital, Paddington, Hospital for Epilepsy and Paralysis, Regent's Park, and the Metropolitan Convalescent Institution; and £25 each to the Provident Surgical Appliance Society.

### Latest Installations in Hospital Equipment.

### WHAT OTHERS ARE DOING.

***** We desire information calculated to keep this column up to date in the interest of institutional workers generally. Secretaries and other executive officers are invited to forward particulars of new installations in any department. In this way a file will be available for all hospital authorities contemplating improvements and anxious to know what other institutions have found effective and modern.**

### HIGH-PRESSURE STERILISERS AND DRESSING WAGONS.

The Surgical Manufacturing Co., of 83-85 Mortimer Street, W., have received an order from the War Office for 120 large-size high-pressure sterilisers and 2,000 drums for the sterilisers. This firm also recently supplied the War Office with 916 dressing wagons and 1,000 aseptic chairs.

### HORTON ASYLUM.

The London County Council Hospitals Committee have provided a number of new beds for Horton Asylum (now renamed the County of London War Hospital), 580 of which were supplied by Messrs. Fisher, Brown and Bayley, Ltd., Lion Works, Lionel Street, Birmingham.

### BRITISH AND FOREIGN SAILORS' SOCIETY.

This Society, which has done so much for the comfort of our brave seamen, is appealing for funds in order that the scope and usefulness of its work may be increased. We are informed that since the declaration of the blockade by Germany the crews, and in many instances the passengers, from no fewer than forty-two ships have been received into the various Sailors' Homes and Institutes conducted by the Society. The rescued seamen have received food and shelter and medical attention, and in not a few cases an entire outfit of clothing has been provided. But this represents only a small proportion of the great work that has been done, and we commend the Society's appeal to our readers. Contributions of money, clothing, etc., should be sent to the Sailors' Palace, Commercial Road, London, E.

RATES OF SUBSCRIPTION: Twelve months, 6/6; Six months, 4/-; Three months, 2/-, post free to any address in the United Kingdom. Abroad, Twelve months, 8/8; Six months, 5/-; Three months, 2/6, post free.—Address The Subscription Dept., "THE HOSPITAL," The Hospital Building, 28/29 Southampton Street, Strand, London, W.C.

By Sir ARBUTHNOT LANE, Bart., M.S.

**OPERATIVE TREATMENT OF FRACTURES.**

*SECOND EDITION. With* 226 *Illustrations.* 10s.

THE MEDICAL PUBLISHING COMPANY, LTD.,
23 Bartholomew Close, E.C.

THE OPERATIVE TREATMENT OF

**CHRONIC INTESTINAL STASIS.**

With additional Chapters by
Drs. JAMES MACKENZIE, JORDAN and MUTCH.

*THIRD EDITION. With* 102 *Illustrations.* 10*s.* 6*d. net.*

J. NISBET & CO., LTD., 22 Berners Street, W.

**CLEFT PALATE AND HARE-LIP.**

With additional Chapters by
Mr. CORTLANDT MACMAHON and Mr. WARWICK JAMES.

*THIRD EDITION. With* 58 *Illustrations.* 10*s.*

THE MEDICAL PUBLISHING COMPANY, LTD.,
23 Bartholomew Close, E.C.

